# Controlled ovarian stimulation should not be preferred for male infertility treated with intrauterine insemination: a retrospective study

**DOI:** 10.1186/s12958-021-00730-3

**Published:** 2021-03-19

**Authors:** Yan Tang, Qian-Dong He, Ting-Ting Zhang, Jing-Jing Wang, Si-Chong Huang, Yun Ye

**Affiliations:** Department of Gynecology and Obstetrics, Centre for Reproductive Medicine, Zhongshan City People’s Hospital, No. 2, Sunwen East Road, Shiqi District, Zhongshan, 528400 Guangdong Province China

**Keywords:** Intrauterine insemination, Controlled ovarian stimulation, Clinical pregnancy, Live birth, Multiple pregnancy

## Abstract

**Background:**

Some studies have stated that intrauterine insemination (IUI) with controlled ovarian stimulation (COS) might increase the pregnancy rate, while others suggest that IUI in the natural cycle (NC) should be the first line of treatment. It remains unclear whether it is necessary to use COS at the same time when IUI is applied to treat isolated male factor infertility. Thus, we aimed to investigate efficacy of IUI with COS for isolated male factor infertility.

**Methods:**

A total of 601 IUI cycles from 307 couples who sought medical care for isolated male factor infertility between January 2010 and February 2020 were divided into two groups: NC-IUI and COS-IUI. The COS-IUI group was further divided into two subgroups according to the number of pre-ovulatory follicles on the day of HCG: cycles with monofollicular development (one follicle group) and cycles with at least two pre-ovulatory follicles (≥ 2 follicles group). The IUI outcomes, including clinical pregnancy, live birth, spontaneous abortion, ectopic pregnancy, and multiple pregnancy rates were compared.

**Results:**

The clinical pregnancy, live birth, spontaneous abortion, and ectopic pregnancy rates were comparable between the NC-IUI and COS-IUI group. Similar results were also observed among the NC-IUI, one follicle, and ≥ 2 follicles groups. However, with respect to the multiple pregnancy rate, a trend toward higher multiple pregnancy rate was observed in the COS-IUI group compared to the NC-IUI group (8.7% vs. 0, *P* = 0.091), and a significant difference was found between the NC-IUI and ≥ 2 follicles group (0 vs. 16.7%, *P* = 0.033).

**Conclusion:**

In COS cycles, especially in those with at least two pre-ovulatory follicles, the multiple pregnancy rate increased without a substantial gain in overall pregnancy rate; thus, COS should not be preferred in IUI for isolated male factor infertility. If COS is required, one stimulated follicle and one healthy baby should be the goal considering the safety of both mothers and foetuses.

**Supplementary Information:**

The online version contains supplementary material available at 10.1186/s12958-021-00730-3.

## Background

Infertility, defined as failure to achieve pregnancy within 12 months of unprotected intercourse or therapeutic donor insemination in women younger than 35 years or within 6 months in women older than 35 years, affects approximately 15% of couples [1]. Male factor infertility is the sole responsible in about 20% of infertile couples and a contributor in another 30 to 40% [2, 3].

Intrauterine insemination (IUI) is an effective and frequently used fertility treatment for couples with male factor and unexplained infertility since it is less invasive, less stressful, more easily accepted by patients, and more cost-effective [4–9]. In IUI, a small volume of prepared semen, removing content which might interfere with fertilisation, such as dead and immotile spermatozoa, debris, white cells, and seminal plasma, is injected directly into the uterine cavity around the expected time of ovulation. The rationale of this procedure is to bypass the cervical barrier, increase the density of normal motile spermatozoa, and bring the spermatozoa closer to the released oocyte [6, 10].

IUI can be performed with or without controlled ovarian stimulation (COS). Some studies stated that IUI with COS might increase the pregnancy rate [11–14], while others suggested that IUI in natural cycle (NC) should be the first-line treatment [4, 15–17]. Thus, it remains unclear if it is necessary to use COS at the same time when IUI is applied to treat isolated male factor infertility. Therefore, we performed a retrospective study to investigate the efficacy of IUI with COS in male patients with infertility. To the best of our knowledge, the present study includes the largest number of couples with male infertility to date in which the outcome of IUI was analysed between NC and COS cycles.

## Methods

### Patients

A retrospective study was performed by reviewing the clinical data of 601 IUI cycles from 307 couples who sought medical care for male infertility at the Center for Reproductive Medicine of Zhongshan City People’s Hospital from January 2010 to February 2020. The inclusion criteria were as follows: (1) couples diagnosed with primary or secondary infertility; (2) the female partner had a normal fertility status, characterised by regular menstrual cycles, normal uterine cavity, and positive post-coital test; and (3) there was at least one patent fallopian tube assessed by hysterosalpingography/transvaginal real-time three-dimensional hysterosalpingo-contrast sonography and/or laparoscopy. Couples were excluded if the female partner had bilateral tubal pathology, endometriosis, irregular cycles, polycystic ovarian syndrome, or other endocrine disorders. Of note, in our centre, before 2014, the diagnosis of male infertility was defined as one or more of subnormal semen variables: sperm concentration < 20 × 10^6^/mL; motility < 50%; normal morphology < 15%, according to the World Health Organisation (WHO) criteria (4th version). Additionally, since 2014, the diagnosis was changed as follows: total sperm < 39 × 10^6^/ejaculation or sperm concentration < 15 × 10^6^/mL; progressive motility < 32%; and normal morphology < 4%, according to the current WHO criteria (5th version). All diagnoses were based on at least two semen analyses. The study protocol was approved by the institutional ethics committee of Zhongshan City People’s Hospital, and informed consent for their clinical data to be used for research purposes was obtained from all participants.

### Ovarian stimulation protocols and follicle monitoring

Ovarian stimulation drugs included clomiphene citrate (CC), letrozole (LE), human menopausal gonadotropin (HMG), and urine follicle-stimulating hormone (uFSH; Livzon, Zhuhai, China). The ovarian stimulation protocols were as follows: (1) CC 50–100 mg/day or LE 2.5–5 mg/day starting from days 3–5 of the menstrual cycle for 5 days; (2) HMG or uFSH 37.5–75 IU/day starting from days 3–5 for a variable duration depending on the response; (3) CC/LE combined with HMG/uFSH: CC 50–100 mg/day or LE 2.5–5 mg/day starting from days 3–5 for 5 days followed by addition of 37.5-75 IU HMG/uFSH for a variable duration depending on the response.

Follicle growth was monitored using transvaginal ultrasonography by gynaecologists in the reproductive medicine centre. In both NC and COS cycles, follicle development was monitored from days 8–10, and then repeated every 2 or 3 days based on the follicle size. Couples were advised to cancel the cycle if more than 3 dominant follicles > 16 mm were present. Ovulation was triggered by administering human chorionic gonadotrophin (hCG) 5000–10,000 IU or triptorelin (Diphereline; Ipsen, Paris, France) 0.1 mg when at least one follicle of 18 mm or more was seen on transvaginal ultrasonography. If endogenous LH surge occurred (more than 20 international units), the trigger was omitted. Insemination was performed 36–40 h later.

### Semen preparation and insemination

On the day of IUI, semen was processed by density-gradient centrifugation or the swim-up method after liquefaction. The pre- and post-wash semen parameters, such as volume, sperm concentration, progressive motility, and normal sperm morphology, were recorded. The volume of the washed semen sample used for insemination was 0.5 mL. IUI was performed by a gynaecologist in a room adjacent to the laboratory. After IUI, the women underwent bed rest for 30 min.

### Luteal phase support and follow-up

Luteal phase support consisting of 20 mg/d dydrogesterone (Duphaston; Abbott, Chicago, IL) was routinely used since the day of ovulation (usually on the day of insemination or the next day). If pregnancy was confirmed on days 14–16 after IUI by measuring serum β-hCG levels, luteal phase support was continued up to 8 weeks of gestation. In women with positive β-hCG, transvaginal ultrasound examination was performed 2 weeks later to confirm clinical pregnancy. The outcomes of clinical pregnancy were subsequently recorded, including spontaneous abortion, ectopic pregnancy, multiple pregnancy, and live birth. Live birth was defined as the birth of an infant after 28 weeks of gestation with postnatal evidence of life.

### Statistical analysis

Statistical analysis was performed using the Statistical Package for Social Sciences (SPSS, version 16.0, for Windows). Fisher’s exact test was used to compare categorical data between the groups. The Mann-Whitney U-test was used to compare continuous variables between groups. Statistical significance was set at *P* < 0.05. According to the study by Cohlen et al. [15], to detect a difference of this magnitude (as opposed to zero hypothesis of no difference), 150 cycles would be needed for each treatment modality with α set at 0.05 and β at 0.20 (power = 80%).

## Results

A total of 307 couples completed 601 IUI cycles. Among them, 265 cycles (194 NC cycles and 71 COS cycles) were completed before 2014, accounting for 44.1%, and the rest (231 NC cycles and 105 COS cycles) were performed in 2014 or after. The mean ± standard deviation (SD) of female age, male age, and duration of infertility were 31.0 ± 4.8, 33.2 ± 5.4, and 2.9 ± 1.9 years, respectively. The mean ± SD total progressive motile sperm counts (TPMSC, volume × sperm concentration × percentage of progressive motile spermatozoa) and the percentage of normal morphology post-wash before 2014 were 19.5 ± 14.7 (× 10^6^) and 8.2 ± 2.1 (%), respectively and 17.6 ± 11.0 (× 10^6^) and 4.6 ± 1.4 (%) in 2014 or after. A total of 75 cycles resulted in pregnancy, and 69 babies were delivered. The clinical pregnancy and live birth rates were 12.5 and 11.3%, respectively. Of the 75 pregnancies, 68 resulted in the birth of a single child (*n* = 67) or twins (*n* = 1), five pregnancies ended in spontaneous abortion, two pregnancies ended in ectopic pregnancy, and there was one case of heterotopic pregnancy who underwent laparoscopic left salpingectomy at 7 weeks of gestation, and delivered a live baby by caesarean section at 35 weeks of gestation. Neither high-order multiples nor ovarian hyperstimulation syndrome occurred.

Table 1 shows the comparison of characteristics of patients and cycles between group NC-IUI and group COS-IUI. Significant differences were seen in the number of dominant follicles ≥16 mm (NC vs. COS: 1.0 ± 0.1 vs. 1.5 ± 0.5, *P* < 0.001) but not in age of the female or male partner, type of infertility, duration of infertility, and endometrial thickness. The TPMSC and the percentages of normal morphology were also comparable between the two groups.

**Table 1:** Characteristics of patients and cycles between the NC-IUI and COS-IUI groups

Characteristics	NC-IUI	COS-IUI	*P* -value
n	425	176	
Female age (years)	31.3 ± 4.8	31.1 ± 4.5	0.701
Male age (years)	33.6 ± 5.7	33.5 ± 5.3	0.935
Type of infertility (n (%))			0.264
Primary	263 (61.9)	118 (67.0)	
Secondary	162 (38.1)	58 (33.0)	
Duration of infertility (years)	2.9 ± 2.0	3.0 ± 2.2	0.648
No. of dominant follicles ≥16 mm	1.0 ± 0.1	1.5 ± 0.5	< 0.001^*^
Endometrium thickness (mm)	9.9 ± 1.5	9.9 ± 1.6	0.971
TPMSC (×10^6^)			
pre-wash	55.8 ± 59.4	53.2 ± 37.8	0.405
post-wash	18.1 ± 13.1	19.0 ± 12.0	0.175
Normal morphology (%)			
pre-wash	4.4 ± 2.0	4.4 ± 2.5	0.442
post-wash	6.2 ± 2.3	6.2 ± 2.9	0.220

*NC* Natural cycle, *COS* Controlled ovarian stimulation, *TPMSC* Total progressive motile sperm count; ^*^ denotes a statistically significant difference (*P* < 0.05)

The pregnancy outcomes between the two groups, including the clinical pregnancy, live birth, spontaneous abortion, and ectopic pregnancy rates, were also comparable. Although a trend toward higher multiple pregnancy rates was observed in the COS-IUI group (8.7% vs. 0), the difference was not statistically significant (*P* = 0.091, Table 2). The COS cycles were further divided into two subgroups according to the number of pre-ovulatory follicles on the day of HCG: cycles with monofollicular development and those with at least two pre-ovulatory follicles. No significant differences were found in the clinical pregnancy, live birth, spontaneous abortion, and ectopic pregnancy rates among the NC-IUI, one follicle, and ≥ 2 follicles groups. However, a significant difference was found between the NC-IUI group and ≥ 2 follicles group in the multiple pregnancy rate (0 vs. 16.7%, *P* = 0.033). The results are presented in Table 2.

**Table 2:** Comparison of pregnancy outcomes between the NC-IUI and COS-IUI groups

	NC-IUI^a^	COS-IUI			*P*-value			
		Total^b^	1 follicle^b1^	≥ 2 follicles^b2^	a vs. b	b1 vs. b2	a vs. b1	a vs. b2
n	425	176	93	83				
Clinical pregnancy (n (%))	52 (12.2)	23^*^ (13.1)	11(11.8)	12^*^ (14.5)	0.787	0.658	1.000	0.588
Live birth (n (%))	47 (11.1)	21^*^ (11.9)	10(10.8)	11^*^ (13.3)	0.778	0.647	1.000	0.572
Spontaneous abortion (n (%))	3 (5.8)	2 (8.7)	1(9.1)	1(8.3)	0.639	1.000	0.546	0.574
Ectopic pregnancy (n (%))	2 (3.7)	1^*^ (4.3)	0	1^*^ (8.3)	1.000	1.000	1.000	0.458
Multiple pregnancy (n (%))	0	2^*^ (8.7)	0	2^*^(16.7)	0.091	0.478	–	0.033^#^

*NC* Natural cycle, *COS* Controlled ovarian stimulation; ^*^ including one heterotopic pregnancy; ^#^denotes a statistically significant difference (*P* < 0.05)

^a^, Group NC-IUI; ^b^, Group COS-IUI; ^b1^, Group 1 follicle; ^b2^, Group ≥2 follicles

To assess whether the semen quality influenced the outcome of either treatment, we divided the study population into three groups: couples with TPSMC < 5 × 10^6^; couples with TPSMC 5–10 × 10^6^; and couples with TPSMC ≥10 × 10^6^. The pregnancy outcomes between the NC-IUI and COS-IUI groups were compared in each subgroup. No significant differences were observed (Table 3). We also compared the characteristics of the patients and cycles in each subgroup. The number of dominant follicles ≥16 mm was significantly higher in the COS-IUI group than in the NC-IUI group in all three subgroups (*P* < 0.001). Other parameters were comparable except for the duration of infertility in subgroup TPSMC 5–10 × 10^6^ (NC vs. COS: 3.3 ± 2.3 vs. 2.9 ± 2.8, *P* = 0.023) (Supplemental Table 1).

**Table 3:** Comparison of pregnancy outcomes between the NC-IUI and COS-IUI groups stratified by TPSMC

Characteristics	TPSMC < 5 × 10^6^			TPSMC 5–10 × 10^6^			TPSMC ≥10 × 10^6^		
	NC	COS	*P*	NC	COS	*P*	NC	COS	*P*
n	36	14		98	37		291	125	
Clinical pregnancy (n (%))	2(5.6)	1(7.1)	1.000	14(14.3)	6(16.2)	0.789	36(12.4)	16(12.8) ^a^	0.873
Live birth (n (%))	2(5.6)	1(7.1)	1.000	13(13.3)	5(13.5)	1.000	32(11.0)	15(12.0) ^a^	0.739
Spontaneous abortion (n (%))	0	0	–	1(7.1)	1(16.7)	0.521	2(5.6)	1(6.2)	1.000
Ectopic pregnancy (n (%))	0	0	–	0	0	–	2(5.6)	1(6.2) ^a^	1.000
Multiple pregnancy (n (%))	0	0	–	0	0	–	0(0)	2(12.5) ^a^	0.09

*IUI* Intrauterine insemination, *NC* Natural cycle, *COS* Controlled ovarian stimulation, *TPMSC* Total progressive motile sperm count; ^a^ including one heterotopic pregnancy

To eliminate the potential influence of repeated cycle data and different diagnostic criteria (WHO 4th version and WHO 5th version) on the results, only the first cycle was included and further divided into two subgroups: before 2014 and from 2014 onwards. In each subgroup, no significant differences were found in pregnancy outcomes between the NC-IUI and COS-IUI groups (Table 4).

**Table 4:** Year-based subgroup analysis of patient characteristics and pregnancy outcomes in the first IUI cycle

Characteristics	Before 2014			From 2014 onwards		
	NC-IUI	COS-IUI	*P* -value	NC-IUI	COS-IUI	*P* -value
n	111	21		146	29	
Female age (years)	30.9 ± 5.1	29.5 ± 3.7	0.245	31.5 ± 4.6	30.0 ± 4.8	0.074
Male age (years)	33.2 ± 5.8	32.8 ± 4.6	0.356	33.4 ± 5.2	33.3 ± 5.8	0.845
Type of infertility (n (%))			1.000			0.683
Primary	80 (72.1)	15 (71.4)		83(56.8)	18(62.1)	
Secondary	31(27.9)	6 (28.6)		63(43.2)	11(37.9)	
Duration of infertility (years)	2.9 ± 1.9	3.5 ± 2.5	0.447	2.8 ± 1.7	2.6 ± 2.4	0.189
No. of dominant follicles ≥16 mm	1.0 ± 0.0	1.5 ± 0.5	0.000	1.0 ± 0.1	1.4 ± 0.5	0.000^*^
Endometrium thickness (mm)	10.3 ± 1.5	10.0 ± 1.6	0.488	9.7 ± 1.4	9.5 ± 1.7	0.534
TPMSC (×10^6^)						
pre-wash	71.6 ± 89.9	58.8 ± 54.4	0.661	47.5 ± 36.7	48.2 ± 33.5	0.804
post-wash	20.4 ± 15.8	16.9 ± 10.0	0.597	16.5 ± 10.6	18.4 ± 12.8	0.696
Normal morphology (%)						
pre-wash	6.0 ± 1.6	5.7 ± 1.8	0.977	3.0 ± 1.2	3.3 ± 1.2	0.203
post-wash	8.2 ± 1.9	7.5 ± 1.7	0.633	4.7 ± 1.5	4.8 ± 1.4	0.508
Clinical pregnancy (n (%))	13 (11.7)	2(9.5)	1.000	19(13.0)	5(17.2)	0.557
Live birth (n (%))	10(9.0)	2(9.5)	1.000	18(12.3)	4(13.8)	0.765
Spontaneous abortion (n (%))	1(7.7)	0	1.000	1(5.3)	1(20.0)	0.380
Ectopic pregnancy (n (%))	2(15.4)	0	1.000	0	0	–
Multiple pregnancy (n (%))	0	0	–	0	0	–

*IUI* Intrauterine insemination, *NC* Natural cycle, *COS* Controlled ovarian stimulation, *TPMSC* Total progressive motile sperm count; ^*^ denotes a statistically significant difference (*P* < 0.05)

## Discussion

Male infertility has become a global health issue. It has been reported that semen counts have declined over the past 50 years [18]. According to the results of Agarwal et al. [19], at least 30 million men worldwide are infertile, with the highest rates seen in Africa and Eastern Europe. IUI has long been considered as the first-line treatment for male infertility. The published pregnancy rates with IUI in NC cycles vary from 0 to 20.5%, while in stimulated cycles this rate varies from 3.9–13.6% per cycle [4] . In our study, the pregnancy and live birth rates in NC cycles were 12.2 and 11.1% per cycle, respectively, while the corresponding values for COS cycles were 13.1 and 11.9%, respectively. Despite the higher rate seen in COS cycles, no statistically significant differences were observed. To assess whether the semen quality influenced the outcome of either treatment, we divided the study population into three groups: couples with TPSMC < 5 × 10^6^; couples with TPSMC 5–10 × 10^6^; and couples with TPSMC ≥10 × 10^6^. Similar results were observed in all subgroups.

It has also been suggested that COS could overcome subtle ovulation disorders that cannot be detected by routine testing [17, 20]; to some extent, multifollicular growth is associated with increased pregnancy rates, and IUI with COS is always preferred.

A 1999 randomised trial [14] showed that couples treated with superovulation had higher pregnancy rates than did couples without stimulation after four IUI cycles (33% vs. 18%). However, it should be noted that the stimulation in this study was intense (150 IU follicle-stimulating hormone as the initial dose), and the multiple pregnancy rates were high, which is unacceptable. In the present study, couples were advised to cancel the cycle if more than three dominant follicles ≥16 mm were present.

Another retrospective cohort study by Huang et al. [11] suggested that, in an IUI programme for unexplained or mild male factor infertility, ovarian stimulation with LE may significantly increase live birth rates while controlling multiple pregnancy rates. In that study, compared with NC (6.2%), live birth rates were significantly higher in IUI cycles stimulated with CC (8.9%), LE (9.4%), and gonadotropins (9.5%). The multiple pregnancy rates in the NC, CC, LE, and gonadotropin groups were 0.7, 4.6, 1.3, and 3.9%, respectively. However, as proposed by those authors, female age, duration of infertility, and first cycle proportion were significantly different among the four groups, which was a limitation of that study.

In addition, Liu et al. [12] concluded that ovarian stimulation in ovulatory women undergoing IUI appeared to have a limited role, with the exception of LE and HMG. In their study, when all ovarian stimulation protocols were combined, no differences in pregnancy or live birth rates were found in ovulatory women. However, further analysis of the impact of the different ovarian stimulation protocols showed that the live birth and pregnancy rates of ovulatory women undergoing IUI treatment stimulated with LE combined with HMG were significantly higher than those in an NC (12.2% vs. 7.6, and 16.8% vs. 9.3%, respectively). However, in that retrospective study, four types of infertility aetiologies (endometriosis, tubo-peritoneum, male factor, and unexplained infertility) were included. It should be noted that the efficacy of ovarian stimulation might differ from infertility aetiologies. As it has been reported the addition of active ovulation management (CC + HCG) increased the pregnancy rate per IUI cycle in unexplained infertility; however, in couples with male infertility, this protocol may not be beneficial [17]. In our study, only the couples with male infertility were included, and we believe the results might be more generalizable.

An increasing number of studies [4, 15–17] reported that IUI with or without COS for male infertility has no significant differences in the pregnancy rate, which is consistent with the results of two recent Cochrane reviews [10, 21]. Furthermore, in our study, to eliminate the potential influence of repeated cycle data and different diagnostic criteria (WHO 4th and 5th versions) on the results, we only included the first cycle and performed a subgroup analysis between cycles before 2014 and from 2014 onwards, which revealed similar results. In addition, the cumulative live birth rates after three IUI cycles with a similar protocol were 23% for NC-IUI and 25% for COS-IUI (*P* = 0.837, data not shown). Therefore, we advocate that for male infertility, IUI with COS did not significantly increase the pregnancy and live birth rates, regardless of the diagnostic criteria for male infertility. Moreover, from the perspective of cost-effectiveness, IUI alone is a more cost-effective approach than IUI with COS [4].

Two patients who received IUI with COS had multiple pregnancies (one twin and one heterotopic pregnancy, both with HMG protocol, and in both two follicles were generated) in our study. A trend toward higher multiple pregnancy rates was observed in the COS-IUI group, when compared to that in the NC-IUI group, but the difference was not statistically significant (*P* = 0.091). However, when we further compared the multiple pregnancy rates between the NC-IUI group and ≥ 2 follicles group, a significant difference was found (0 vs. 16.7%, *P* = 0.033). Few studies have focused on reporting multiple pregnancies of IUI with or without COS in male infertility.

A meta-analysis [22] included 14 studies reporting on 11,599 IUI cycles in patients with various types of subfertility and showed that the pregnancy rates increased from 8.4 to 15% with COS when multifollicular growth was achieved as compared to monofollicular stimulation, and the multiple pregnancy rates increased from 3.7 to 17% per conceived cycle. Compared with monofollicular growth, when stimulating two, three, and four follicles, the pregnancy rates increased by 5, 8, and 8%, while the risk of multiple pregnancies increased by 6, 14, and 10%, respectively. Those authors advocated that IUI with COS should not aim for more than two stimulated follicles; one stimulated follicle should be the goal if safety is the primary concern, whereas two follicles may be acceptable after careful patient counselling. Recently, data from another systematic review and meta-analysis [23] did not support the routine use of gonadotropins for ovarian stimulation in IUI for women with unexplained infertility, as the increased pregnancy rate is associated with a larger increased risk of multiple gestations.

Additionally, Evans et al. [24] also reported that the mean clinical pregnancy rate per IUI ranged from 13.0% with one mature follicle to 19.6% with five mature follicles. The pregnancy rate increased modestly with the presence of each additional mature follicle (adjusted odds ratio: two follicles vs. one: 1.3, 95% CI 1.2–1.4; three follicles vs. one: 1.4, 95% CI 1.3–1.5; four follicles vs. one: 1.5, 95% CI 1.4–1.7; and five follicles vs. one: 1.6, 95% CI 1.4–1.9). However, a greater significantly increased risk of multiple pregnancies was seen with the presence of each additional mature follicle (adjusted odds ratio: two follicles vs. one: 3.5, 95% CI 2.7–4.4; three follicles vs. one: 5.6, 95% CI 4.4–7.1; four follicles vs. one: 7.2, 95% CI 5.6–9.4, and five follicles vs. one: 8.6, 95% CI 6.2–11.8). There was only a 1.9% increase in singleton pregnancies per IUI regardless of mature follicle number (from 12.4% with one follicle to 14.3% with five follicles).

Compared with singleton pregnancies, multiple pregnancies are associated with many pregnancy complications, which is unacceptable [25]. Therefore, the aim of fertility treatment is shifting from focusing on the pregnancy rate to the birth of healthy singletons [26].

Our study had several limitations. First, the retrospective nature of our study might have led to potential inherent bias. In addition, we only included cycles in which IUI was performed. Cycles with more than three dominant follicles ≥16 mm were cancelled in all groups and were not included in this study. The data from these cycles may favour IUI with COS. Finally, the time span of this study was 10 years, which means the diagnostic criteria about male infertility changed, since the WHO manual (5th version) is widely used as a source of standard methodology for semen collection, analysis, and preparation. However, other elements of the IUI practice did not change. We also performed an analysis stratified for different criteria to eliminate the potential influence on the results as much as possible.

## Conclusions

To the best of our knowledge, the present study included the largest number of couples with male infertility to date in which the pregnancy outcomes of IUI were analysed between NC and COS cycles. The findings of the current study suggest that for male infertility, COS in IUI should not be preferred, since in cycles with COS, especially in those with at least two pre-ovulatory follicle cycles, the multiple pregnancy rate increased without substantial gain in the overall pregnancy rate. If COS is required, one stimulated follicle and one healthy baby should be the goal considering the safety of both mothers and foetuses.

## Supplementary Information

**Additional file 1: Supplemental Table 1.** Characteristics of patients and cycles between the NC-IUI and COS-IUI groups stratified by TPSMC.

**Additional file 2: Supplemental Figure 1.** Consort diagram. IUI, intrauterine insemination; NC, natural cycle; COS, controlled ovarian stimulation; ^*^including one heterotopic pregnancy; ^#^ two patients gave birth to a single child in the first cycle and planned to have a second child; ^##^ one patient gave birth to a single child in the fourth cycle and planned to have a second child.

## Data Availability

The data sets used and/or analysed during the current study are available from the corresponding author on reasonable request.

## Abbreviations

CC: Clomiphene citrate
COS: Controlled ovarian stimulation
HCG: Human chorionic gonadotrophin
HMG: Human menopausal gonadotropin
IUI: Intrauterine insemination
LE: Letrozole
NC: Natural cycle
TPMSC: Total progressive motile sperm count
uFSH: Urine follicle-stimulating hormone
WHO: World Health Organisation
