# Supplementary material for: Controlled ovarian stimulation should not be preferred for male infertility treated with intrauterine insemination: a retrospective study
Source: Reprod Biol Endocrinol. 2021 Mar 19;19:45. doi: 10.1186/s12958-021-00730-3 (PMC7977560; doi:10.1186/s12958-021-00730-3)
Supplement: Supplementary file 1 — Additional file 1: Supplemental Table 1. Characteristics of patients and cycles between the NC-IUI and COS-IUI groups stratified by TPSMC. [file 12958_2021_730_MOESM1_ESM.docx]

**Supplemental Table 1: Characteristics of patients and cycles between the NC-IUI and COS-IUI groups stratified by TPSMC**

| Characteristics | TPSMC < 5 × 10^6^ | | | |  | TPSMC 5-10 × 10^6^ | | |  | TPSMC ≥ 10 × 10^6^ | | |
| --- | --- | --- | --- | --- | --- | --- | --- | --- | --- | --- | --- | --- |
|  | NC | COS | | *P* |  | NC | COS | *P* |  | NC | COS | *P* |
| n | 36 | 14 |  | |  | 98 | 37 |  |  | 291 | 125 |  |
| Female age (years) | 29.0±4.2 | 29.2±4.5 | 0.983 | |  | 31.3±4.6 | 30.5±5.0 | 0.318 |  | 31.6±4.9 | 31.5±4.3 | 0.962 |
| Male age (years) | 31.0±4.6 | 33.4±6.9 | 0.473 | |  | 34.0±6.1 | 33.4±5.5 | 0.757 |  | 33.8±5.7 | 33.5±5.0 | 0.765 |
| Type of infertility (n (%)) |  |  | 0.746 | |  |  |  | 0.838 |  |  |  | 0.101 |
| Primary | 25(69.4) | 9(64.3) |  | |  | 68(69.4) | 25(67.6) |  |  | 170(58.4) | 84(67.2) |  |
| Secondary | 11(30.6) | 5(35.7) |  | |  | 30(30.6) | 12(32.4) |  |  | 121(41.6) | 41(32.8) |  |
| Duration of infertility (years) | 3.0±2.0 | 2.8±1.6 | 0.767 | |  | 3.3±2.3 | 2.9±2.8 | 0.023* |  | 2.8±1.9 | 3.0±2.1 | 0.400 |
| No. of dominant follicles ≥16 mm | 1.0±0.2 | 1.6±0.5 | <0.001* | |  | 1.0±0.1 | 1.6±0.6 | <0.001* |  | 1.0±0.1 | 1.4±0.5 | <0.001* |
| Endometrium thickness (mm) | 9.6±1.4 | 9.9±1.7 | 0.440 | |  | 9.9±1.7 | 10.1±1.7 | 0.331 |  | 9.9±1.4 | 9.8±1.6 | 0.359 |
| TPMSC ( ×106) |  |  |  | |  |  |  |  |  |  |  |  |
| pre-wash | 11.9±5.1 | 12.2±6.3 | 1.000 | |  | 21.4±12.9 | 23.4±20.0 | 0.842 |  | 72.8±64.6 | 66.7±35.5 | 0.655 |
| post-wash | 4.0±0.8 | 3.8±1.0 | 0.484 | |  | 7.2±1.4 | 7.3±1.6 | 0.728 |  | 23.6±12.4 | 24.2±10.4 | 0.141 |
| Normal morphology ( %) |  |  |  | |  |  |  |  |  |  |  |  |
| pre-wash | 4.0±1.7 | 3.7±2.5 | 0.359 | |  | 4.4±1.8 | 4.1±2.0 | 0.275 |  | 4.4±2.1 | 4.6±2.6 | 0.951 |
| post-wash | 5.8±2.1 | 6.0±3.3 | 0.810 | |  | 6.2±2.1 | 5.5±2.5 | 0.068 |  | 6.3±2.4 | 6.4±3.0 | 0.698 |

IUI, intrauterine insemination; NC, natural cycle; COS, controlled ovarian stimulation; TPMSC, total progressive motile sperm count; *denotes a statistically significant difference (*P*<0.05).
