# Supplementary material for: Controlled ovarian stimulation should not be preferred for male infertility treated with intrauterine insemination: a retrospective study
Source: Reprod Biol Endocrinol. 2021 Mar 19;19:45. doi: 10.1186/s12958-021-00730-3 (PMC7977560; doi:10.1186/s12958-021-00730-3)
Supplement: Supplementary file 2 — Additional file 2: Supplemental Figure 1. Consort diagram. IUI, intrauterine insemination; NC, natural cycle; COS, controlled ovarian stimulation; *including one heterotopic pregnancy; # two patients gave birth to a single child in the first cycle and planned to have a second child; ## one patient gave birth to a single child in the fourth cycle and planned to have a second child. [file 12958_2021_730_MOESM2_ESM.docx]

**Supplemental Figure 1:**

The first cycle

301 patients (cycles) were included

NC-IUI (n=257)

COS-IUI (n=50)

Live birth: 28 patients

Spontaneous abortion：2 patients

Ectopic pregnancy：2 patients

Live birth: 6 patients

Spontaneous abortion：1 patient

Drop-out：90 patients

-out：98 patients

The second cycle

185 patients (cycles) ^#^ were included

NC-IUI (n=117)

COS-IUI (n=68)

Live birth: 13 patients

Spontaneous abortion：1 patient

Live birth: 8 patients

Spontaneous abortion：1 patient

Ectopic pregnancy^*^：1 patient

Multiple pregnancy^*^：2 patients

Drop-out：81 patients

-out：98 patients

The third cycle

83 patients (cycles) were included

COS-IUI (n=43)

Live birth: 5 patients

Live birth: 6 patients

NC-IUI (n=40)

Drop-out： 52 patients

-out：98 patients

The fourth cycle

20 patients (cycles) were included

COS-IUI (n=12)

Live birth: 1 patient

Live birth: 1 patient

NC-IUI (n=8)

Drop-out：15 patients

-out：98 patients

The fifth cycle

4 patients (cycles) ^##^ were included

COS-IUI (n=3)

No pregnancy

No pregnancy

NC-IUI (n=1)

Drop-out：2 patients

-out：98 patients

The sixth cycle

2 patients (cycles) were included

No pregnancy

NC-IUI (n=2)

Figure 1: Consort diagram. IUI, intrauterine insemination; NC, natural cycle; COS, controlled ovarian stimulation; ^*^including one heterotopic pregnancy; ^#^ two patients gave birth to a single child in the first cycle and planned to have a second child; ^##^ one patient gave birth to a single child in the fourth cycle and planned to have a second child.
